# A Novel Vermiculite/TiO_2_ Composite: Synergistic Mechanism of Enhanced Photocatalysis towards Organic Pollutant Removal

**DOI:** 10.3390/molecules28176398

**Published:** 2023-09-01

**Authors:** Lin Han, Xiaoju Yue, Liying Wen, Mingqing Zhang, Shifeng Wang

**Affiliations:** 1 Innovation Laboratory of Materials for Energy and Environment Technologies, Institute of Oxygen Supply, College of Science, Tibet University, Lhasa 850000, China; 2 Key Laboratory of Cosmic Rays (Tibet University), Ministry of Education, Lhasa 850000, China; 3 Fujian Quanzhou Peninsula Materials Co., Ltd., Quanzhou 362000, China; 4 Aimoli (Hebei) Technology Co., Ltd., Shijiazhuang 050000, China

**Keywords:** composite material, heterojunction, adsorption, photocatalysis, bifunctional

## Abstract

There has been increasing concern over water pollution, which poses a threat to human life and health. Absorption by low-cost absorbents is considered to be a cost-effective and efficient route. However, the non-reusability of absorbents greatly limits their applications. In this study, a novel vermiculite/TiO_2_ composite combining the inexpensive absorbent with the commonly used photocatalyst was firstly synthesized via the sol-gel method. On the one hand, the organic pollutants are absorbed by vermiculite and then decomposed through the photocatalysis process, enabling the next round of absorption and creating an absorption–decomposition reusable cycle. On the other hand, the modulation effect of optical and electronic structure on the prepared TiO_2_ photocatalyst by the vermiculite incorporation could significantly improve the photocatalytic activity and eventually enhance the aforementioned cyclic degradation capacity. The layer-structured vermiculite (Vt) supports a uniform coverage of TiO_2_ at an optimized ratio, providing an optimal adsorption environment and contact area between the photocatalyst and methylene blue (MB) molecules. Vt/TiO_2_ heterojunction is formed with Si-O-Ti bonding, at which electrons transfer from Vt to TiO_2_, enriching electron density in TiO_2_ and favoring its photocatalytic activity. Furthermore, the incorporation of Vt increases the light absorption of TiO_2_ in the visible range by narrowing the optical band gap to 1.98 eV, which could promote the generation of photo-excited carriers. In addition, PL measurements revealed that the carrier recombination is substantially suppressed, and the charge separation and migration are greatly enhanced by a factor of 3. As a result, the decomposition rate of MB is substantially increased 5.3-fold, which is ascribed to the synergistic effects of the elevated photocatalysis and the large absorption capacity governed by the chemisorption mechanism of the intra-particle diffusion. These results pave the way for composite design towards efficient, economical, and pragmatic water pollution treatment.

## 1. Introduction

Water pollution has long been a global concern, posing significant threats to human life and health. The treatment of industrial wastewater has emerged as an increasingly pressing issue. Among the organic pollutants present, methylene blue (MB), commonly utilized in chemical indicators, dyes, biological stains, and pharmaceuticals, has gained particular attention [1]. Its high solubility in water, substantial loss during production and use, and the persistent residue it leaves in the environment make its complete removal through conventional treatment methods challenging [2,3,4]. MB was categorized by the World Health Organization in 2017 as a carcinogen, highlighting its potential to damage human organs and cause nerve damage upon long-term exposure. Consequently, numerous studies have focused on developing efficient methods to remove MB from water. These studies have explored various adsorbents such as plant fibers, biochar, fruit peel, and clay [5,6,7]. Among these options, clay minerals offer a promising choice for wastewater treatment owing to their abundance, affordability, and high removal rates [8,9,10]. Among the various types of clay adsorbents, the 2:1-type stabilized layered silicate mineral known as vermiculite (Vt) has garnered preference among researchers for water pollution treatment. Vt primarily operates through electrostatic adsorption and ion exchange. Owing to its abundance and low cost, Vt can be used in high quantities in wastewater treatment [11,12]. Therefore, recent research has predominantly concentrated on the modification of Vt with various organics to enhance its maximum adsorption capacity [13,14,15]. However, these synthesis approaches involving organic compounds often produce secondary pollutants, leading to contamination during recycling. Moreover, the adsorption capacity would reach a saturation point, at which organic substances will no longer be absorbed.

In addition to adsorption methods, photocatalytic degradation has also gained considerable traction in wastewater treatment, particularly for eliminating toxic and hazardous compounds [16,17]. Titanium dioxide (TiO_2_) has been extensively studied as a popular catalyst due to its high reaction speed, low energy consumption, high catalytic efficiency, and durability [18,19]. However, TiO_2_ nanoparticles possess several drawbacks, such as large energy band gap of the intrinsic material (>3.2 eV), extreme agglomeration, and phase transitions under heat treatment, which affect their photocatalytic efficiency and hinder their application.

Herein, a novel Vt/TiO_2_ composite was synthesized in this work with combined adsorption and photo-degradation capabilities. Using the sol-gel method, the composite was prepared through hydrolytic condensation reactions of titanium isopropoxide (TIPT) on the Vt surface and interlayer region. TiO_2_ was introduced into Vt at different mass ratios, forming a Vt/TiO_2_ heterojunction with Si-O-Ti bonds at the interfaces. While mechanism studies indicate that the adsorption of Vt is primarily chemisorption-dominated, the electron transfer from Vt to TiO_2_ does not contribute significantly to the adsorption kinetics. As a result, the presence of loaded TiO_2_ nanoparticles partially occupies the adsorption sites in Vt, reducing the composite’s adsorption capacity. However, the Vt/TiO_2_ heterostructure is crucial in narrowing the optical band gap, enabling enhancements in sunlight capture and generation of photoexcited carriers. Moreover, the composite can effectively inhibit the photogenerated charge carriers from recombination and facilitates the separation and migration of electron–hole pairs. These factors greatly enhance the photocatalytic performance of the composite by a factor of 5.3. The composite demonstrates dual functionality, exhibiting high adsorption capacity for organic pollutants while simultaneously decomposing the organic molecules into H_2_O and CO_2_ through enhanced photocatalytic activity. This unique combination enables continuous adsorption and establishes an adsorption-photodegradation recyclable approach for organic pollutant removal. The present study integrated adsorption and photocatalysis in a bifunctional composite to remove hazardous organic pollutants. This approach is promising for future material design aimed at addressing environmental issues.

## 2. Results and Discussion

### 2.1. Structural and Photoelectronic Properties

**Figure 1:**
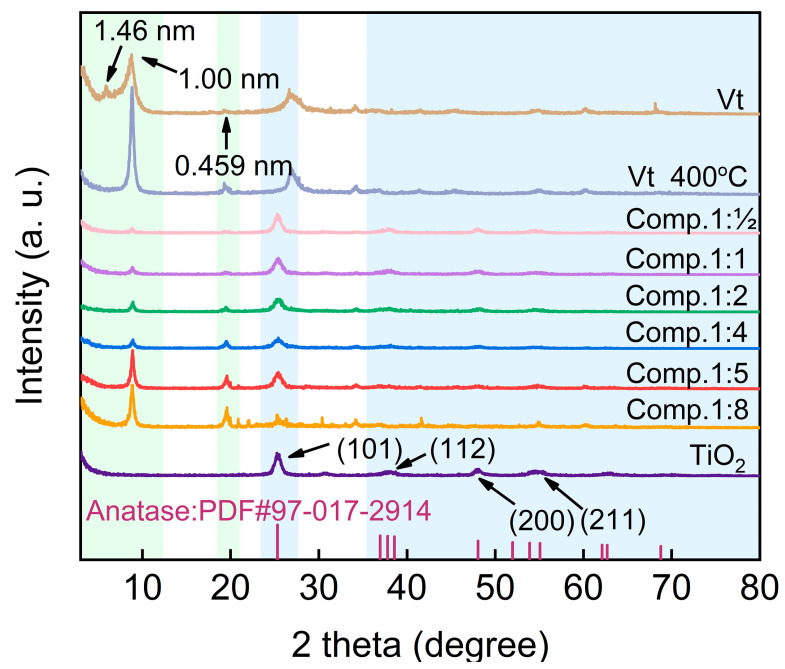
XRD patterns of the as-synthesized Vt/TiO_2_ composite.

Figure 1 presents the XRD patterns of the synthesized Vt/TiO_2_ composite. The top line displays the pure Vt diffraction peaks associated with the characteristic spacing of 1.46 nm (consisting of physisorbed water molecules), 1.00 nm (containing exchangeable cations), and 0.459 nm. After calcining at 400 °C, as demonstrated by the second upper line in Figure 1, the peak representing water molecules through physical adsorption (1.46 nm) disappears, while the peaks corresponding to lattice distance of 1.00 nm and 0.459 nm become sharp and intense, which is probably due to the improved crystallinity of SiO_2_ in Vt after calcination. Thus, the peaks associated with 1.00 nm and 0.456 nm are used for Vt determination hereafter. The bottom line in Figure 1 represents the pure TiO_2_ calcined at 400 °C, in which peaks located at 2θ = 25.3°, 38.7°, 48.1° and 54.4° are clearly observed, corresponding to the (101), (112), (200), and (211) diffraction planes of TiO_2_ in anatase phase, respectively. The other patterns show the Vt/TiO_2_ composite calcined at the same temperature of 400 °C with varying TiO_2_/Vt mass ratio. In the Figure 1 analysis, representative peaks were distinctly identified by employing color-coded backgrounds: Vt was highlighted in green, while TiO_2_ was marked in blue. Peaks both from Vt and anatase TiO_2_ are detected in the composite, manifesting the successful combination of Vt and TiO_2_. Furthermore, the peak denoting the characteristic spacing of 1.00 nm is gradually strengthened with the increasing Vt content not surprisingly. While the ratio of TiO_2_ to Vt exceeds 1:8, the signal from TiO_2_ is almost undetectable, which consequently weakens the photocatalytic performance of the composite material.

**Figure 2:**
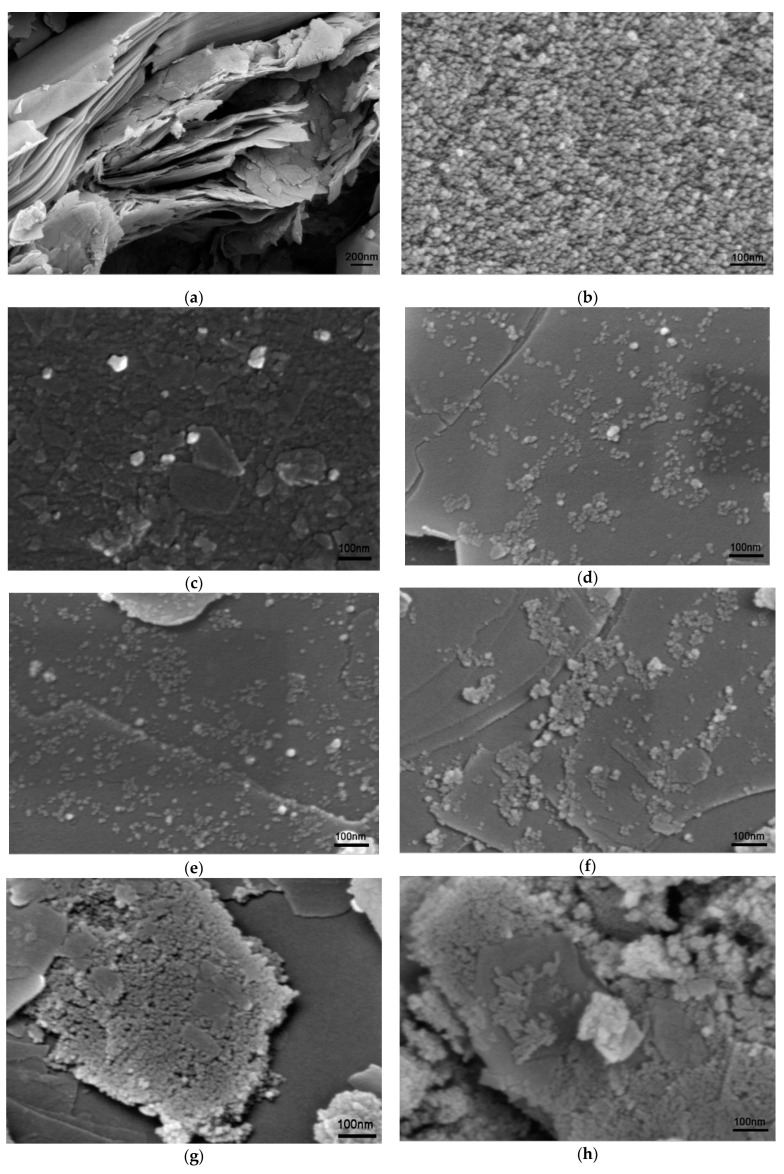
SEM morphologies of samples (**a**) Vt; (**b**) TiO_2_; (**c**) Comp.1:8; (**d**) Comp.1:5; (**e**) Comp.1:4; (**f**) Comp.1:2; (**g**) Comp.1:1; (**h**) Comp.1:0.5.

SEM images, as shown in Figure 2, are employed to characterize the morphology of the prepared Vt, TiO_2_, and their composite. Figure 2a presents the morphology of the prepared Vt, which exhibits a typical layered structure. In contrast, TiO_2_ has an aggregated granular morphology, as shown in Figure 2b. As demonstrated in Figure 2c–h, with the increase in relative TiO_2_ content, the number of small TiO_2_ particles on the surface of Vt, or the coverage of TiO_2_ onto Vt, is increasing to the point of aggregation. When the TiO_2_:Vt ratio reaches 1:1, as displayed in Figure 2g, the Vt surface is extensively covered with TiO_2_ nanoparticles, while it seems to reach TiO_2_ loading saturation as the TiO_2_:Vt ratio continuously rises to 1:0.5, since the excessive TiO_2_ nanoparticles break away from the Vt surface and form aggregates.

**Figure 3:**
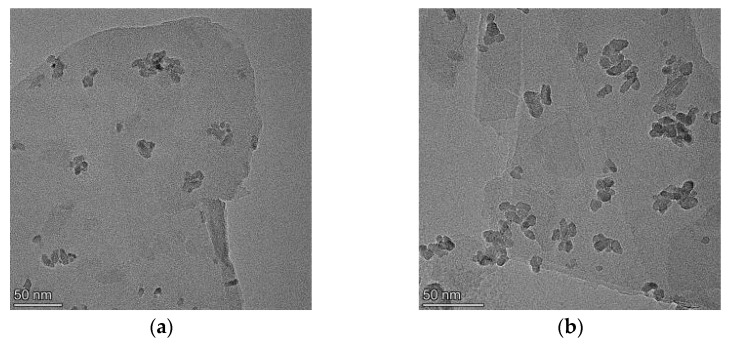

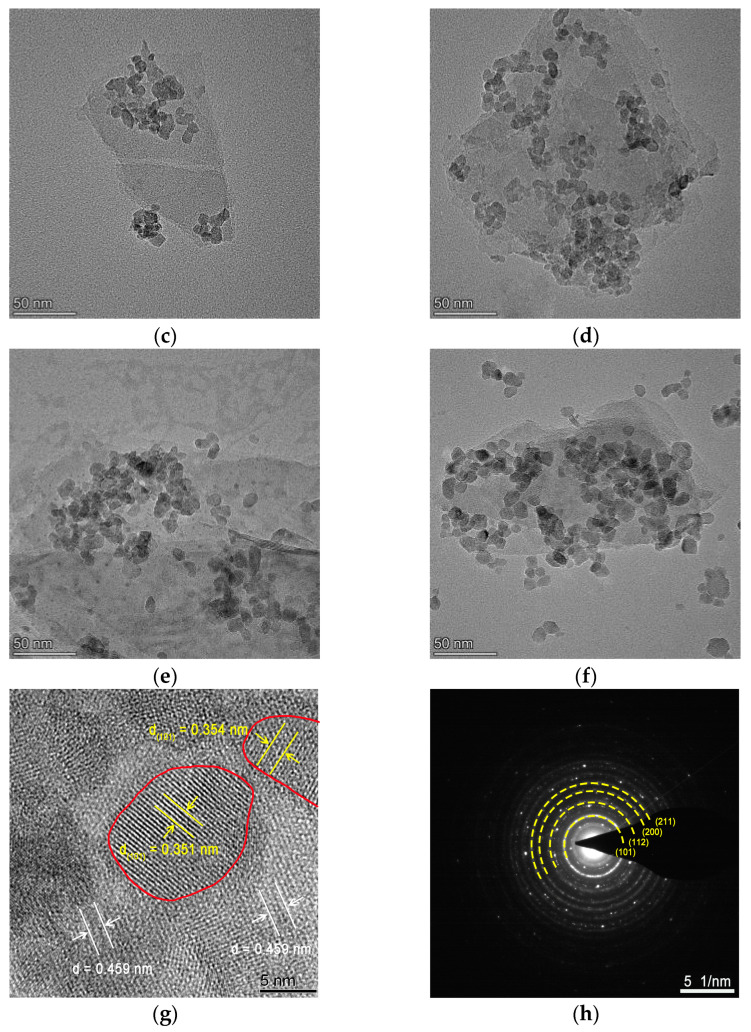
FE-TEM images of the composites: (**a**) Comp.1:8; (**b**) Comp.1:5; (**c**) Comp.1:4; (**d**) Comp.1:2; (**e**) Comp.1:1; (**f**) Comp.1:0.5; (**g**) lattice fringes of Comp.1:5; (**h**) SAED pattern of Comp.1:5.

The distribution of TiO_2_ onto Vt is more visually evident in the TEM images of the composites (Figure 3). It is clearly seen from Figure 3a–f that the density of TiO_2_ nanoparticles as indicated by the darker grains per unit area on the thinner Vt flakes grows as the TiO_2_ content in the composite increases. When a certain ratio is reached, the TiO_2_ nanoparticles start to agglomerate (Figure 3e) or to get over-loaded (Figure 3f). Upon TiO_2_ saturation as shown in Figure 3f, it is observed that the excessive particles own a higher agglomeration tendency than those loaded on the Vt surface, which is further confirmed via the BET analysis of the composites listed in Table 1. The specific surface area of the composites is reasonably high compared to that of the pure Vt. The presence of TiO_2_ has a positive effect on the enlargement of the specific surface area of the composites. The higher the relative content of TiO_2_ in the composite, the larger the specific surface area that can be obtained. When the ratio of TiO_2_ to Vt reaches 1:2 (Comp.1:2), the surface area of the composites no longer increases, which is ascribed to the particle agglomeration caused by the overloading of TiO_2_. Further increasing the TiO_2_ content gives rise to a drop in surface area (Comp.1:0.5), which is due to large-scale agglomeration of excessive TiO_2_ nanoparticles outside the Vt. A high-resolution TEM image of this composite (Figure 3g) provides the strongest visual evidence of Vt/TiO_2_ heterostructure formation, in which two different systems of lattice fringes are distinctly observed. The characteristic spacing of 0.351 nm in the image center is assigned to the (101) lattice plane of anatase TiO_2_, while the fringe distance of 0.459 nm around the TiO_2_ particle originates from Vt, which is determined via the XRD analysis above. As indicated by the red circles in Figure 3g, the prepared TiO_2_ nanoparticles with the size of about 10 nm are incorporated onto Vt layer, building heterostructure between them. The Vt/TiO_2_ heterojunction formed would facilitate the electron transfer as well as the photo-generated carriers separation, endowing the composite material a superior photocatalytic performance. Figure 3h presents the selected area electron diffraction (SAED) pattern of the composite (Comp.1:5), exhibiting intermittent circles and corresponding to the (101), (112), (200), and (211) diffraction planes of TiO_2_, respectively, which are well consistent with the XRD measurements and suggest the polycrystalline nature of the prepared TiO_2_.

**Figure 4:**
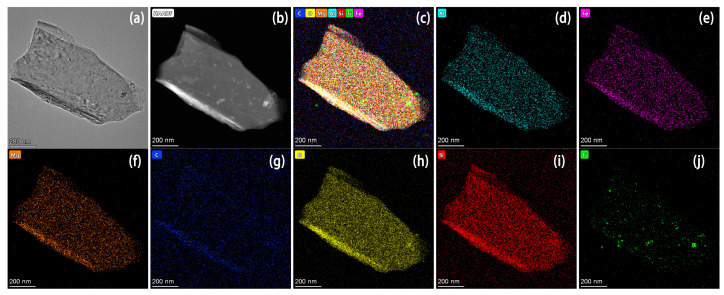
HAADF images of the composite material (Comp.1:5). (**a**) TEM image, (**b**) HAADF image, (**c**) all-element distribution map, (**d**) Al, (**e**) Fe, (**f**) Mg, (**g**) C, (**h**) O, (**i**) Si, and (**j**) Ti elemental distribution maps of Comp.1:5.

High-angle annular dark field (HAADF) images are shown in Figure 4. It can clearly be seen that the Si, Mg, Al, Fe, C, and O elements are uniformly distributed, confirming the substrate in Figure 4b being Vt, while the Ti element is distributed separately, whose position is well coincident with the spot on the substrate, suggesting the TiO_2_ nanoparticles covering onto Vt.

**Figure 5:**
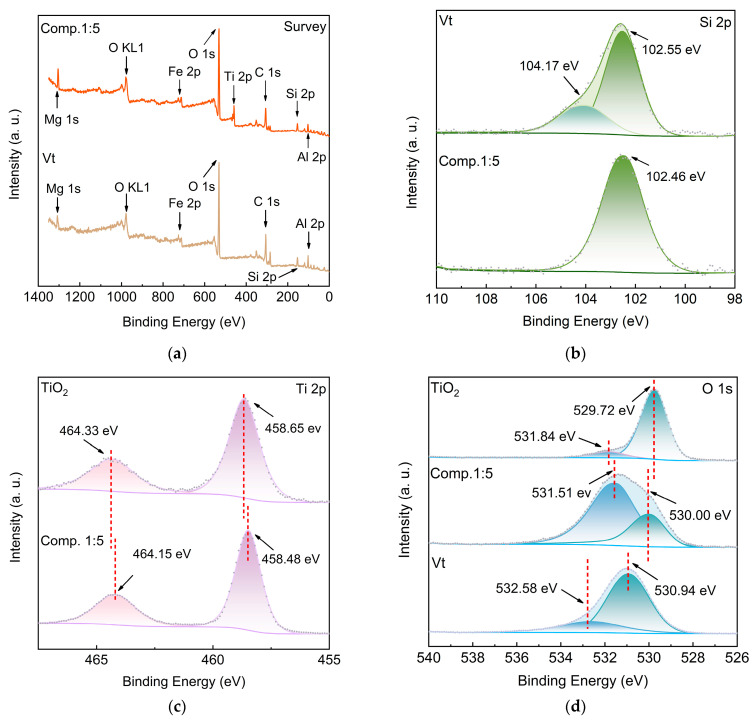

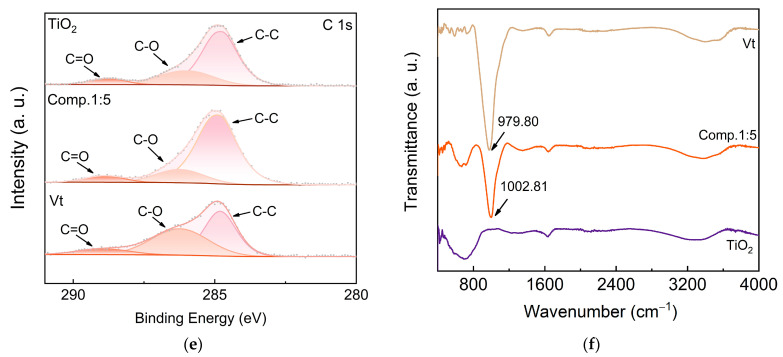
Electronic structure analysis. (**a**–**e**) XPS spectra of Vt, TiO_2_, and their composite: survey scans; Si 2p scans; Ti 2p scans; O 1s scans; C 1s scans; (**f**) FTIR spectra.

XPS measurements were carried out on Vt, TiO_2_ and their composite material (Comp.1:5) to reveal the chemical composition and the surface electronic structure (Figure 5). In the survey scans, as shown in Figure 5a, the peaks representing elements Si, Mg, Al, Fe, O, C appear in both Vt and composite samples, confirming the constituent components of Vt. The presence of the additional Ti peak in the composite sample verifies the successful incorporation of TiO_2_ onto Vt. The high resolution Si 2p spectra of Vt in Figure 5b can be deconvoluted into two peaks, a primary peak at 102.55 eV and a secondary peak at 104.17 eV, corresponding to the Si-O bonds of aluminosilicate structure and the Si bonds connected with the surface hydroxyl groups in Vt, respectively. Compared with Vt, only the Si characteristic peak at 102.46 eV in the SiO_2_ tetrahedra is reflected in the composite, and the Si bonds associated with the surface hydroxyl groups at higher binding energy disappear in the composite. It is speculated that the disappearance of Si-O peak at higher binding energy is attributed to either the reaction of Si-OH on the surface with TiO_2_ to form Si-O-Ti bonds or the loss of hydroxyl groups due to the high temperature calcination of the composite [20,21]. As shown in Figure 5c, the peaks at 458.65 eV and 464.33 eV correspond to Ti^4+^ 2p_3/2_ and Ti^4+^ 2p_1/2_ in TiO_2_, respectively [22]. In comparison with pure TiO_2_, the Ti 2p peaks in the composite display a small shift of about 0.18 eV towards lower binding energy, indicating that the valence state of Ti element in TiO_2_ is reduced. Figure 5d shows the high resolution O 1s spectra of TiO_2_, Vt and their composite. The O 1s spectrum of TiO_2_ can be resolved into two components, a main peak at 529.72 eV and a broad peak at 531.84 eV, which are assigned to the lattice O_2_- bond and a very small amount of oxygen vacancy in TiO_2_, respectively. In contrast, the O 1s spectrum of Vt can also be fitted into two peaks at 530.94 eV and 532.58 eV, which are assigned to the O-metal bonds of aluminosilicate and surface adsorbed water molecules, respectively [23]. When TiO_2_ nanoparticles are covered onto the Vt substrate, it is worth noting that both the lattice O^2−^ bonds in TiO_2_ and the O-metal bonds of aluminosilicate in Vt exhibit appreciably positive shifts of 0.28 eV and 0.57 eV, respectively, which reveals that the electrons of the O site are attracted and transferred from Vt to TiO_2_, enriching electron density in TiO_2_ and favoring its photocatalytic activity. The incorporation of TiO_2_ onto Vt promotes the electron-donating ability of O sites in Vt, and results in higher valence states, enabling the composite to generate strong electronic interactions between Vt and TiO_2_ nanoparticles. Carbon is ubiquitous and will contaminate surfaces of all samples during the XPS analysis. Therefore, the carbon C 1s peak at 284.8 eV is commonly used as a reference for charge correction. Figure 5e illustrates the C 1s peaks of surface-contaminated TiO_2_, Vt, and their composite. All spectra can be fitted into three peaks located at 284.8 eV, 286.2 eV, and 288.9 eV, which are associated with C-C, C-O, and C=O bonds, respectively. It is worth noting that the content of C-O in Vt is much higher than that in TiO_2_ and the composite, suggesting a reduction of C-O bonds after the incorporation of TiO_2_ into the composite, and thus indicating that there is a considerable number of C-O bonds formed through water molecules’ participation in the Vt break, which are rearranged to Vt-O-Ti bonds after the TiO_2_ addition and calcination. This analysis is in good agreement with those of the Si and O spectrum variations.

As shown in Figure 5f, FTIR analysis was further conducted to verify the band states of the composite. In the bottom curve of pure TiO_2_, absorption peaks can be detected at 500-900 cm^−1^, originating from the O-Ti-O bond [21]. The sharp peak in the Vt spectrum at 979.8 cm^−1^ can be attributed to Si-O stretching vibration mode. In comparison, the above characteristic peaks show up in the middle curve of the Vt/TiO_2_ composite. However, the band of Si-O exhibits a red shift to 1002.8 cm^−1^, suggesting the reconstruction of Si-O-Ti bonds with the incorporation of TiO_2_ through hydroxyl groups [21]. FTIR analysis further verifies the successful preparation of Vt/TiO_2_ composite, but also confirms the formation of Si-O-Ti bonds and a heterojunction between Vt and TiO_2_ nanoparticles.

**Figure 6:**
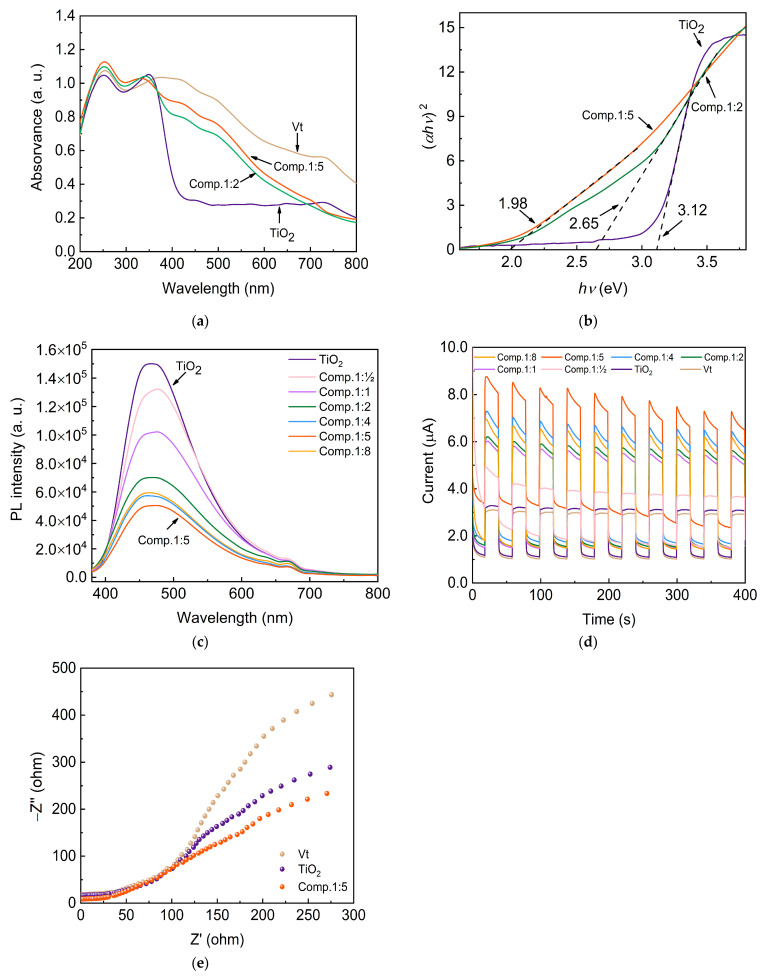
Photoresponse analysis of the Vt/TiO_2_ composites. (**a**) UV-vis absorption spectra; (**b**) Tauc plots; (**c**) PL spectra; (**d**) transient photocurrent response; (**e**) Nyquist plots.

The built Vt/TiO_2_ heterostructure and the strong electronic interactions between Vt and TiO_2_ could be expected to accelerate the transfer and separation of the photo-generated carriers. Figure 6a displays the UV-vis absorption spectra of TiO_2_, Vt, Comp.1:5, and Comp.1:2. The synthesized TiO_2_ nanoparticles have high absorption in the UV region (the absorption edge is about 390 nm) and basically no absorption in the visible region. Vt has relatively strong absorption in the full spectrum. In contrast, the absorption ability of the composites in the visible range is lower than that of pure Vt and gradually decreases with the wavelength. However, the combination of Vt and TiO_2_ still extends the absorption bandwidth substantially to the visible range. The enhanced visible light response of the composite can be expected to capture more sunlight and generate more photo-induced carriers, thus boosting the photocatalytic performance.

The energy band gap of a semiconductor can be deduced from the Tauc plot: ${\left(\alpha h\upsilon \right)}^{1/n}=A(h\upsilon -E_{g})$, where *α* is the absorption coefficient, *h* is the Planck’s constant, *ν* the photon’s frequency, *A* is a proportionality constant, $E_{g}$ is the optical energy band gap, and the value of the exponent donotes the nature of the electronic transition [21,24,25]. As shown in Figure 6b, the band gap of TiO_2_ was determined to be 3.12, very close to the theoretical value of 3.2 eV, while those of the Comp.1:2 and Comp.1:5 were calculated to be 2.65 eV and 1.98 eV, respectively. Consequently, the Comp.1:5 sample is expected to harvest more sunlight than the Comp.1:2, thus generating more electron–hole pairs and delivering better photocatalytic capacity. PL spectra were employed to characterize the recombination and migration properties of photogenerated electrons in the composite materials (Figure 6c). It was observed that the pure TiO_2_ nanoparticles show the strongest PL peak, while the peak intensity gradually decreases with the increase in the addition amount of Vt until the ratio of TiO_2_ to Vt reaches 1:5. It is worth noting that pure Vt does not have a photoresponse. Therefore, excessive Vt in the composite material will greatly reduce the overall photo-generated carriers and also expose long distance for the carrier migration. Stronger fluorescence suggests an easier recombination of the photogenerated electron–hole pairs of the material, making it difficult for the electron–hole pairs to migrate to the surface of the catalyst to participate in the photocatalytic redox reaction, which is not conducive to the performance of photocatalysis [26]. Hence, Comp.1:5 sample is expected to own the best photocatalytic activities due to an enhanced charge carrier extraction and suppressed electron–hole recombination. The transient photocurrent response was conducted to determine the transfer and generation of the photoexcited charge carriers [27]. As shown in Figure 6d, the photocurrent response of Comp.1:5 was improved by a factor of 3 in comparison with pure TiO_2_, which demonstrates that Vt/TiO_2_ heterojunction possesses enhanced charge separation under visible light irradiation, leading to greater photocatalytic performance. The Nyquist plots of EIS measurements in Figure 6e reveal the charge transfer resistance of Vt, TiO_2_, and their composite. Generally speaking, a semicircle represents the charge transfer characteristic, and a smaller arc radius suggests more efficient separation of a photogenerated electron–hole pair and a faster interface charge migration process [27]. It can clearly be seen that the Comp.1:5 sample exhibits the smallest semicircle, indicating that it possesses the lowest charge transfer resistance and the most efficient separation of photoexcited charge carriers.

### 2.2. Photocatalytic Experiments

**Figure 7:**
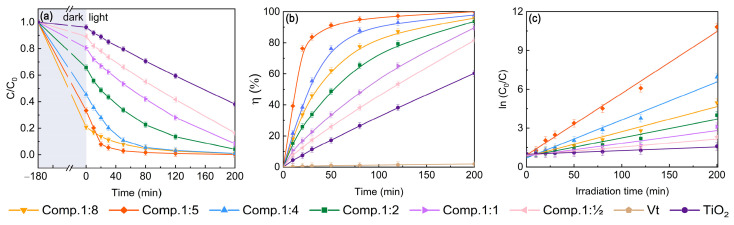
Removal rate analysis of the composites: (**a**) adsorption rate; (**b**) photo-degradation rate; (**c**) kinetic fitting curves.

Methylene blue (MB) was chosen as the pollutant representative for the adsorption and photodegradation tests. A preliminary investigation of the adsorption capacity of the composite materials is demonstrated in Figure 7a. The series of Vt/TiO_2_ composites (1:8, 1:5, 1:4, 1:2, 1:1, 1:0.5) and TiO_2_ achieved their adsorption equilibrium of MB under dark condition for 180 min at the rates of 79.17%, 66.67%, 54.54%, 34.21%, 19.36%, 10.71%, and 3.85%, respectively. This variation is consistent with the microscopic morphology observed via SEM and TEM, and the BET surface area measurements. It is obvious that the absorption capacity of the composite is significantly affected by the different doping levels of TiO_2_. Hence, separate and independent studies on the adsorption and photo-degradation mechanisms of the composites are necessary.

To better understand the photocatalytic performance of different composites, the initial concentration of MB for the samples was adjusted to guarantee the MB concentration at the same level at the very beginning of photocatalysis. At this point, the composite reached adsorption equilibrium and no longer had any adsorption capacity. The TiO_2_ content of 0.5 g/L was maintained in all MB solutions, meaning that the content of photocatalytic substance remained constant. As shown in Figure 7b, the photo-degradation efficiency of the composites after 50 min irradiation exposure first increased gradually from 25.93% to 91.24% as the relative TiO_2_ content in the composites decreased to 1:5. It is worth noting that compared with the synthesized pure TiO_2_ (17.20%), the photo-degradation efficiency of the composite (Comp.1:5) was highly boosted by a factor of 5.3, due to the synergistic effects of the Vt/TiO_2_ heterojunction. Further reducing the relative TiO_2_ content (1:8) results in poor abilities of photogenerated charge separation and transfer, thus lowering the photocatalytic activity (62.12%). The Vt/TiO_2_ composites obtained better photocatalytic capacity than pure TiO_2_. The apparent rate constant (k) calculated from a first-order linear fit of the data on the basis of the results of photodegradation starting at the same concentration is shown in Figure 7c and Table 2. The maximum k value of the composite was 0.48 min^−1^, which was 16 times higher than that of pure TiO_2_, indicating that the Vt/TiO_2_ heterojunction greatly improved the reaction kinetics of TiO_2_.

The improvement in photocatalytic activity and reaction kinetics of the composites can be attributed to the following factors: (i) the skeletal effect of layer-structured Vt on TiO_2_ eases the aggregation of particles and increases the contact area and collision frequency with MB molecules; (ii) Vt/TiO_2_ heterostructure lowers the band gap to capture more sunlight and generate more electron–hole pairs, accelerates the charge separation and transport, and promotes the photocatalytic redox reaction [28]; (iii) Vt donates electrons to TiO_2_ when they are combined, enabling the composite more active to the MB degradation reactions.

### 2.3. Adsorption Mechanism Studies

**Figure 8:**
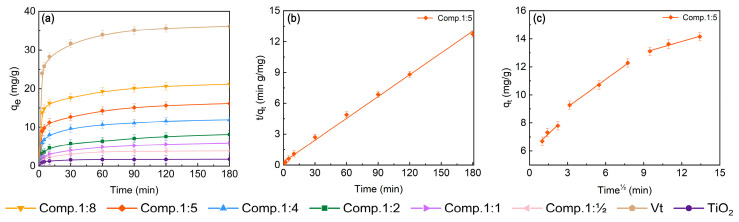
Adsorption kinetics analysis: (**a**) equilibrium adsorption capacity for TiO_2_, Vt and their composites; (**b**) pseudo-secondary model and (**c**) intraparticle model of Comp.1:5 on the adsorption performance.

The determination of the adsorption properties of the composites provides a measure of MB loading, diffusion and access to the photocatalytic sites, which can help us to observe the trend of the absorption and kinetic rate of MB by the TiO_2_/Vt composite photocatalyst. The results are shown in Figure 8a, where it can be observed that MB is quickly absorbed by Vt at the early stage and then gets saturated at around 120 min. The composites show a similar adsorption trend as Vt. The series of Vt/TiO_2_ composites (1:8, 1:5, 1:4, 1:2, 1:1, 1:0.5) and TiO_2_ achieve their equilibrium adsorption capacity of MB under dark conditions for 180 min at 21.15 mg/g, 16.15 mg/g, 11.95 mg/g, 8.15 mg/g, 5.92 mg/g, 3.91 mg/g, and 1.76 mg/g, respectively. Compared to the equilibrium adsorption of pure Vt (36.15 mg/g), with the increase of the relative TiO_2_ content in the composite, the equilibrium adsorption amount decreases, which is ascribed to the coverage of TiO_2_ onto Vt partially occupying adsorption sites on the Vt surface and hindering the adsorption capacity of Vt. The dependence of the adsorption kinetics of MB on duration in the dual-function purification systems is shown in Figure 8. To understand the adsorption kinetics in depth, pseudo-first order (PFO), pseudo-second-order (PSO), and intra particle diffusion (IPD) models are adopted (Table 3) [12]. As shown in Figure 8b and Table 3, the R^2^ fitted to the PSO is higher than 0.99, indicating that the adsorption process is more consistent with the pseudo-second-order. Additionally, the values of q_e,cal_ and q_e,exp_ are close to each other, which further illustrates the applicability of the proposed secondary kinetics. It suggests that the adsorption of composites is dominated by chemisorption [29]. The resulting plot in Figure 8c of the internal diffusion fit shows 3 linear partitions, demonstrating the involvement of intra-particle diffusion, where the first stage indicates the diffusion process on the outer surface. The second stage is the process of intra-ion diffusion, where the particle diffusion rate is the controlling step. From the data in Table 3, it can be seen that the high R^2^ values in the second stage indicate the important role of intra-particle diffusion in the MB adsorption process. The third stage is the equilibrium stage, where the less favorable value of k_id_ represents the intraparticle diffusion process gradually slowing down during this stage as the adsorbate concentration decreases.

**Figure 9:**
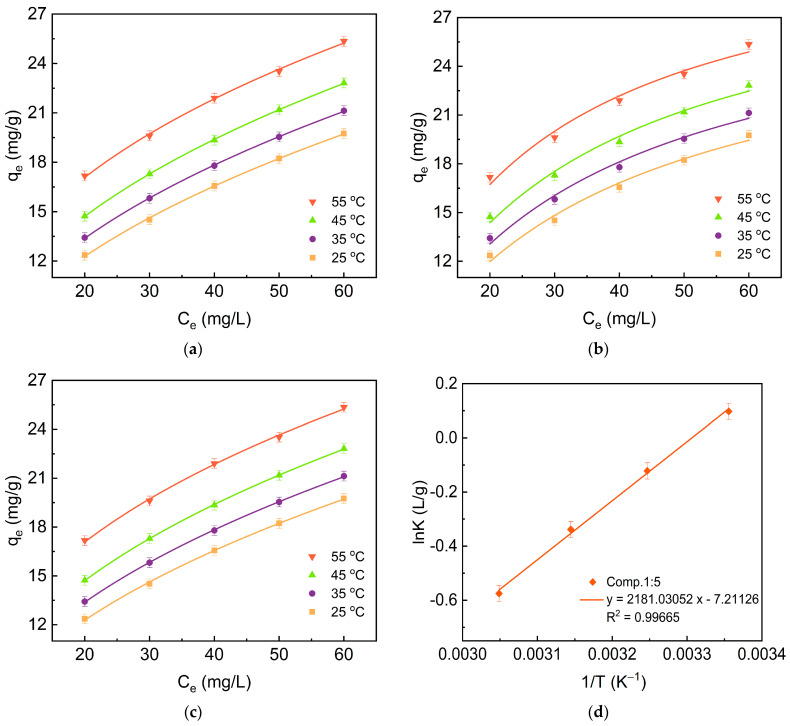
Effect of concentration and temperature on the sorption performance. (**a**) Freundlich model; (**b**) Langmuir model; (**c**) Redlich–Peterson model; (**d**) Thermodynamic curve.

As shown in Figure 9a, the adsorption tends to increase with increasing adsorbate concentration and temperature, with a maximum adsorption of 25.35 mg/g (C_0,MB_ = 60mg/g, T = 55 °C). As shown in Table 4, the Freundlich isotherm is more applicable to the adsorption process of the composite, Comp.1:5, than the Langmuir model because of its higher value of all R^2^, indicating that the adsorbate molecules are easily bound to the heterogeneous adsorption sites in a multilayer arrangement [30]. This can be explained by the fact that MB, initially adsorbed on the surface of the composite due to the benzene ring in its own structure and the functional groups, can generate some π–π interactions with MB in the solution, as well as X-H...π interactions, which eventually form a π–π stacking, causing the MB molecules in the interlayer stacking to adopt a multilayer arrangement. This can also explain why the catalytic effect of Comp.1:8 is reduced in a dense accumulation of many MB molecules in the vicinity of a catalytic site, which ultimately leads to a reduction in the photocatalytic effect of Comp.1:8.

The calculated thermodynamic parameters are listed in Table 5 and Figure 9d. The Gibbs free energy (G) of the adsorption process becomes positive and the adsorption capacity of Comp.1:5 increases with the increase of temperature, suggesting a heat absorption process. The negative value of Δ*S^o^* for the adsorption process indicates that the chaos of the system declines as the adsorption process proceeds.

Adsorption results show that the composites are chemisorption-driven and the adsorption process involves intraparticle diffusion. The adsorbed MB molecules show a multilayer arrangement. The adsorption capacity increases with the temperature growth and the system disorder increases as adsorption proceeds. Although the addition of TiO_2_ into Vt does not help the enhancement in overall adsorption capacity for organic pollutants, the Si-O-Ti-bound Vt/TiO_2_ heterostructure elevates the photocatalytic performance through several aspects of improvement. Thus, the designed bifunctional (or multi-functional) composite can effectively achieve a “adsorption -> photo-degradation -> re-adsorption -> re-photo-degradation” cyclic and recyclable route. Experimentally, it was found that when the ratio of TiO_2_ to Vt was 1:5 (Comp.1:5), the removal rate system reached the best equilibrium state, and MB molecules adsorbed near the photocatalytic site were rapidly decomposed, launching a new cycle of adsorption-photocatalysis procedure.

**Figure 10:**
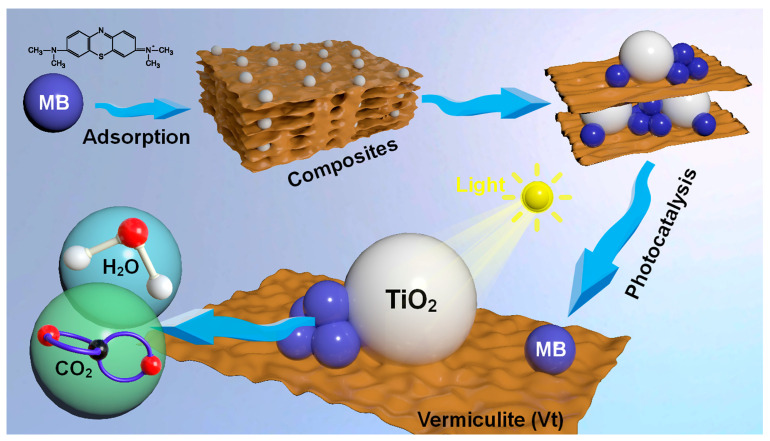
MB structural formula and its degradation mechanism.

As illustrated in Figure 10, MB molecules are initially adsorbed onto the surface and within the interlayers of the composite material, facilitated by the adsorption abilities of Vt, and subsequently decomposed into H_2_O and CO_2_ via the photocatalysis process of TiO_2_. Analyses suggest that the absorption process is chemisorption-dominated with the involvement of intraparticle diffusion. The enhanced photocatalytic ability of TiO_2_ arising from optical and electronic structure regulation with Vt could substantially accelerate the decomposition of the MB absorbed and then leave space for the dissociative MB molecules outside the composite, in turn promoting the attraction and absorption of fresh MB molecules. Vt effectively modulates the optical and electronic structure of TiO_2_, enhancing the photodegradation efficiency of MB. Simultaneously, the decomposed MB facilitates the absorption of fresh MB, forming an “absorption-photodegradation” synergetic process towards an extraordinarily high efficiency of overall removal of pollutants compared to both conventional absorbents and photocatalysts.

## 3. Materials and Methods

### 3.1. Materials

Vermiculite (Vt) (AlFeMgO_3_Si), isopropyl titanate (C_12_H_28_0_4_Ti, 95%), and isopropyl alcohol (C_3_H_8_0, AR, >99.5%), the raw materials for TiO_2_ synthesis, and MB, were all purchased from Shanghai Maclean Biochemical Technology Co., Ltd. All chemicals were used directly without further purification.

### 3.2. Synthesis of TiO_2_/Vt Composites

The synthesis of the composite involved adding Vt to the TiO_2_ synthesis. One gram of Vt was added to the TiO_2_ precursor solution and allowed to undergo complete hydrolysis. The mixture was stirred for 3 h, and the resulting gel was dried in an oven at 60 °C for 12 h. The dried product was ground, sieved (200 mesh), and calcined. By varying the amount of isopropyl titanate while maintaining the calcination temperature (400 °C) constant, composites with different TiO_2_ to Vt mass ratios were obtained. The composites were named as Comp.1:8, Comp.1:5, Comp.1:4, Comp.1:2, Comp.1:1, and Comp.1:0.5, corresponding to TiO_2_:Vt ratios of 1:8, 1:5, 1:4, 1:2, 1:1, and 1:0.5, respectively.

### 3.3. Characterization

The crystal structures of Vt, TiO_2_, and the composites were characterized via a Bruker D8 Advance X-ray diffractometer (XRD), using a Cu target as the radiation source (wavelength 1.5406 Å) at an operating voltage of 40 kV and current of 40 mA. Scanning electron microscopy (SEM) was employed to probe the surface morphology, which was performed on a Quanta 200F electron microscope, while transmission electron microscopy (TEM) was employed to study the microstructure, which was recorded on a FEI Tecnai G2 F30 microscope operating at 200~300 kV. The elemental compositions and electronic states were determined via X-ray photoelectron spectroscopy (XPS), recorded on a Thermo Fisher 250Xi X-ray photoelectron spectrometer. FTIR spectroscopy was carried out on a Nicolet IS10 spectrometer while the light absorption spectra were conducted on a UV-3600 Plus spectrophotometer. Photoluminescence (PL) spectra was recorded on a FL31000 fluorescence spectrophotometer. Electrochemical impedance spectroscopy (EIS) was carried out on a CHI-760E electrochemical workstation with the frequency ranging between 0.01 Hz and 100 kHz.

The methods for material synthesis, along with a comprehensive description of the experimental approaches, are detailed in the Supplementary Material.

## 4. Conclusions

This study synthesized a novel Vt/TiO_2_ composite using the sol-gel method and high-temperature calcination. The Vt component exhibited a layered structure, while the TiO_2_ nanoparticles were synthesized in the anatase phase and distributed onto the Vt layers. Microstructure investigation via TEM revealed that the heterostructure built between Vt and TiO_2_, whereas FTIR spectra indicated the Si-O-Ti bonds formed at the interface of the Vt/TiO_2_ heterojunction, between which strong electronic interactions and electron transfer from Vt to TiO_2_ occurred. Mechanism studies on adsorption demonstrated that the adsorption process in the Vt/TiO_2_ composite is primarily chemisorption-dominated and influenced by temperature. However, the electron environment in the composite did not significantly contribute to the chemisorption process but affected the overall adsorption capacity to some extent. This is attributed to the coverage of TiO_2_ on the Vt surface, which partially occupies the adsorption sites. Nevertheless, incorporating Vt into TiO_2_ resulted in a narrower optical band gap, enabling the composite to effectively harness sunlight and generate more photoexcited charge carriers. Furthermore, the Vt/TiO_2_ heterojunction strongly inhibited the recombination of photogenerated electron–hole pairs by a factor of 3, facilitating the separation and migration of charge carriers and ultimately enhancing the photocatalytic activities.

In comparison to prior research, the Vt/TiO_2_ composites synthesized in the current study have adeptly integrated the adsorption function of Vt with the enhanced photocatalytic performance of TiO_2_. Distinct from the functionalized vermiculite in existing models, the Vt/TiO_2_ composites introduced a novel adsorption-photodegradation cycle, streamlining the removal of organic pollutants in water. With the inclusion of the enhanced photocatalytic properties of TiO_2_, a swift cycle of adsorption-photocatalysis-re-adsorption can be achieved under optimal loading conditions. This addresses the challenge of functional failure after adsorption saturation and the secondary pollution arising from the pollutants release while recycling vermiculite. Additionally, the Vt/TiO_2_ heterojunction formed in the composite effective regulates the optical and electronic structures of the photocatalytic, leading to an enhanced photocatalytic decomposition efficiency and contributing more to the overall removal rate of hazardous pollutants through a synergistic effect.

These composites have demonstrated efficacy in the elimination of organic pollutants, as evidenced by the removal of MB. The adsorbed MB molecules typically formed a multilayered arrangement. An increasing trend in adsorption capacity was observed with rising temperature, peaking at 25.35 mg/g under conditions of C_0_, MB = 60 mg/g and T = 55 °C. Notably, when the composite was governed by a Vt to TiO_2_ ratio of 1:5, the overall degradation rate of MB reached an impressive 91.24% in a mere 50 min, which demonstrates a 5.3-fold increase compared with pure TiO_2_. Should superior-performing photocatalysts be incorporated atop vermiculite, enhanced and expedited removal of organic pollutants from aquatic environments could potentially be achieved. In light of these findings, the composite establishes an “adsorption-photodegradation” cyclic route towards an extraordinarily high efficiency of overall removal for pollutants compared to both conventional absorbents and photocatalysts, positioning it as a compelling solution for cost-effective and highly efficient wastewater treatment through novel multifunctional material design. In future work, more detailed and in-depth studies on the effects of electronic environment variations at the interfaces of the heterojunction on the chemical adsorption and bonding states between the functional groups are required to further improve the absorption capacity and thus, the removal ability.

## Figures and Tables

**Table 1:** BET analysis of Vt, TiO_2_ and their composites.

Samples	SBET (m^2^ g^−1^)	DP (nm)
Vt	18.90	12.58
TiO_2_	103.50	7.99
Comp.1:8	57.45	5.46
Comp.1:5	85.09	7.77
Comp.1:4	114.54	7.38
Comp.1:2	119.21	7.55
Comp.1:1	119.98	8.11
Comp.1:0.5	114.94	7.66

**Table 2:** First order linear fit correlation data for materials.

Photocatalyst	Comp.1:8	Comp.1:5	Comp.1:4	Comp.1:2	Comp.1:1	Comp.1:0.5	TiO_2_
k	0.19	0.48	0.30	0.14	0.09	0.06	0.03
R^2^	0.9696	0.9913	0.9794	0.9413	0.8958	0.9101	0.9716

**Table 3:** Kinetic parameters of MB adsorption on composites.

Adsorbent	q_e,exp_	Pseudo-First-Order			Pseudo-Second-Order				Intra-Particle Diffusion		
		q_e,cal_	k_1_ (10^−2^)	R^2^	q_e,cal_	k_2_ (10^−2^)	k_2_q_e_	R^2^	k_id_	C	R^2^
Comp.1:5	14.15	7.65	−1.07	0.9415	14.14	7.07	0.25	0.9978	0.85	5.96	0.93305
									0.66	7.16	0.99787
									0.26	10.71	0.96184

**Table 4:** Adsorption isotherm constants for MB adsorption on the composite (Comp.1:5) in the adsorption system.

Adsorbent	Temperature	Freundlich			Langmuir			Redlich Peterson			
		K_f_	n	R^2^	Q_max_	K_L_	R^2^	a	b	g	R^2^
Comp.1:5	25 °C	3.38	2.32	0.9992	28.26	0.037	0.9841	580.92	171.61	0.57	0.9988
	35 °C	3.87	2.42	0.9998	29.54	0.039	0.9852	454.42	116.92	0.59	0.9997
	45 °C	4.46	2.51	0.9999	31.29	0.042	0.9857	353.43	78.89	0.61	0.9998
	55 °C	5.89	2.81	0.9982	32.92	0.052	0.9775	1090.87	184.93	0.65	0.9973

**Table 5:** Thermodynamic constants for MB adsorption on the composite Comp.1:5 in the adsorption system.

Adsorbents	$\Delta G^{o}(kJ{mol}^{-1})$				$\Delta S^{o}(JmolK^{-1})$	$\Delta H^{o}(kJ{mol}^{-1})$
	T = 298 K	T = 308 K	T = 318 K	T = 328 K		
Comp.1:5	2.15	2.22	2.29	2.36	−7.21	2.18

## Data Availability

The data are contained within the article.

## Supplementary Materials

The following supporting information can be downloaded at: https://www.mdpi.com/article/10.3390/molecules28176398/s1.
