# Supplementary material for: A Novel Vermiculite/TiO2 Composite: Synergistic Mechanism of Enhanced Photocatalysis towards Organic Pollutant Removal
Source: Molecules. 2023 Sep 1;28(17):6398. doi: 10.3390/molecules28176398 (PMC10489128; doi:10.3390/molecules28176398)
Supplement: Supplementary file 1 [file molecules-28-06398-s001.zip › molecules-2584444-supplementary.pdf]

molecules

MDPI

Supporting Information for Molecules

A Novel Vermiculite/TiO_2 Composite: Synergistic Mechanism of Enhanced Photocatalysis towards Organic Pollutant Removal

Lin Han [1,2], Xiaoju Yue [1,2], Liying Wen [1,2], Mingqing Zhang [1,2] and Shifeng Wang [1,2,3,4,*]

1 Innovation Laboratory of Materials for Energy and Environment Technologies, Institute of Oxygen Supply, College of Science, Tibet University, Lhasa 850000, China
2 Key Laboratory of Cosmic Rays (Tibet University), Ministry of Education, Lhasa 850000, China
3 Fujian Quanzhou Peninsula Materials Co., Ltd., Quanzhou 362000, China
4 Aimoli (Hebei) Technology Co., Ltd.; Shijiazhuang 050000, China
* Correspondence: wsf@utibet.edu.cn

(2 Pages)

Synthesis of TiO_2: Pure TiO_2 was synthesized using the sol-gel method. Initially, 8 mL of isopropyl titanate was mixed with 20 mL of isopropyl alcohol. Deionized water was added to achieve complete hydrolysis, and the mixture was stirred for 3 h at room temperature (25 °C). After stirring, the gel was left in a cool place for 8 h, then dried at 60 °C. The dried product was then ground and sieved through a 200-mesh sieve. Finally, the obtained powder was calcined in a muffle furnace at 400 °C for 4 h to obtain pure TiO2 for subsequent experiments.

Adsorption experiments: All adsorption experiments were conducted in a dark room environment, and intermittent adsorption was used to evaluate the adsorption performance of Vt, TiO_2, and the composites. To analyze the influencing factors and the adsorption mechanism, 0.03 g of the composites were mixed with 30 mL of MB solution, and samples were taken at different time intervals. The changes in MB concentration were measured using UV-vis spectroscopy with a maximum absorbance of 664 nm, according to the Beer–Lambert law. The adsorption amount q_t (mg/g) at time t (in min) was calculated using Equation (S1), where C_0 represents the initial MB concentration, C_t represents the MB concentration (mg/L) at time t, V represents the volume of the solution (L), and m represents the mass of the adsorbent used (g).

$$q_{t,MB}=(C_0\text{-}C_t)\frac{V}{m} \tag{S1}$$

Photocatalytic degradation experiments: The mixture was first adsorbed in the dark for 180 min in a dark room environment. Subsequently, solutions with the same initial concentrations, after dark adsorption, were exposed to a 300 W xenon lamp, and the concentrations were measured via intermittent sampling. All composites were maintained at a TiO_2 concentration of 0.5 g/L in the MB solution during the photocatalytic experiments. MB samples were centrifuged at each time interval, and the transparent top layer was removed. The absorbance of the samples was measured at 664 nm. The photocatalytic degradation rate of MB (η) was calculated using Equation (S2), where C_0 represents C_0 the initial concentration of MB (mg/L) and C_t denotes the concentration of MB after t min of light irradiation (mg/L).

$$\eta_{MB}=C_0\text{-}\frac{C_t}{C_0} \tag{S2}$$

The fitted equations:

$$\ln(q_e - q_t) = \ln q_e - k_1 t \tag{S3}$$

$$\frac{t}{q_t} = \frac{1}{k_2 q_e^2} + \frac{1}{q_e t} \tag{S4}$$

$$q_t = k_{id} t^{1/2} + C \tag{S5}$$

- Pseudo-first-order (PFO): Equation (S3) where q_t and q_e are the adsorption capacities (mg/g) of the composite (Comp.1:5) at time t and adsorption equilibrium, respectively, and k_1 is the rate constant (min^{-1}). $\ln(q_e - q_t)$ is plotted against t, and the corresponding slopes and intercepts, k_1 and q_e, respectively, are listed in Table 3.
- Pseudo-second-order (PSO)： Equation (S4) where q_t is the amount of MB adsorbed at time t (mg/g), q_e is the adsorption capacity at equilibrium (mg/g) and k_2 is the rate constant (g $mg^{-1} min^{-1}$). k_2 and q_e can be obtained by plotting $\frac{t}{q_t}$ against t to obtain a linear line.

  Intra particle diffusion (IPD):Equation (S5) where k_{id} (mg g^{-1} $min^{-1/2}$) is the rate constant of the internal diffusion model; C is the thickness of the boundary layer; and the values of k_{id} and C are obtained by plotting q_t against $t^{1/2}$, as shown in Table 3.

Thermodynamic equation for adsorption: The adsorption thermodynamic parameters were calculated from batch adsorption experiments performed at different temperatures. The specific equations are given as follows:

$$K = C_0 - \frac{C_e}{C_e} \tag{S6}$$

$$\ln K = \frac{\Delta S^o}{R} - \frac{\Delta H^o}{RT} \tag{S7}$$

$$\Delta G^o = \Delta H^o - T\Delta S^o \tag{S8}$$

- where K is the adsorption equilibrium constant, T is the absolute temperature (K), and R = 8.314 J/mol. lnK is plotted against 1/T (Figure 9d), and the slope and intercept of the obtained lines correspond to ΔH° and ΔS°, respectively. The calculated thermodynamic parameters are listed in Table 5. The enthalpy of the adsorption process becomes positive and the adsorption capacity of Comp.1:5 increases with the increase of temperature, which indicates that the adsorption process is a heat absorption process. The negative value of ΔS° for the adsorption process indicates that the chaos of the system increases as the adsorption process proceeds.
